# Molecular insights into fatty acid metabolism reprogramming: Targeting metabolic vulnerabilities in hematological malignancies

**DOI:** 10.1016/j.gendis.2026.102118

**Published:** 2026-03-03

**Authors:** Yue Li, Tiange Lu, Xiangxiang Zhou

**Affiliations:** a Department of Hematology, Shandong Provincial Hospital Affiliated to Shandong First Medical University, Jinan, Shandong 250021, China; b National Clinical Research Center for Hematologic Diseases, The First Affiliated Hospital of Soochow University, Suzhou, Jiangsu 251006, China

**Keywords:** Fatty acid metabolism, Hematological malignancies, Metabolic reprogramming, Metabolic vulnerability, Therapeutic targets, Tumor microenvironment

## Abstract

Lipid metabolism reprogramming is the key mechanism for tumor cells to meet the material and energy required for rapid proliferation. This process involves the comprehensive remodeling of fatty acids, cholesterol, and phospholipid metabolic networks, and drives malignant transformation and disease progression by synergistically regulating the core links of lipid synthesis, uptake, transport, oxidation, and storage. Among them, the reprogramming of fatty acid metabolism plays a key carcinogenic driving role, and its abnormal regulation runs through the whole process of tumor development. Hematological malignancies are characterized by rapid proliferation and high metabolic demands, and rely on the lipid-rich bone marrow microenvironment. For this reason, tumor cells establish unique metabolic adaptability by reconstructing the key metabolic pathways of fatty acids to resist stress such as hypoxia and nutritional deficiency. This metabolic reorganization not only enhances the proliferation and survival advantages but also enhances stemness, promotes chemotherapy resistance, and disrupts signaling pathways, thus constituting a critical basis for disease progression. Targeting the dysregulated fatty acid metabolism has become a highly promising treatment direction, providing new possibilities for the precise risk stratification and improvement of therapeutic effect in different subtypes of hematological malignancies. This review systematically summarizes and critiques the recent important findings in this field. By integrating the framework of metabolic vulnerability and disease heterogeneity, it deeply analyzes its molecular mechanism and biological function, and comments on current and emerging metabolic targeted interventions, aiming to provide a new perspective for the development of precision medicine based on metabolic dependence.

## Introduction

Metabolic reprogramming stands as a defining hallmark of cancer, enabling malignant cells to rewire their energetic and biosynthetic pathways to sustain continuous proliferation and survival. Among the various metabolic alterations, lipid metabolism has evolved from a mere bioenergetic supplier into a pivotal regulatory node that drives the malignant phenotype. Serving as structural components of cellular membranes, precursors for signaling molecules, and modulators of redox homeostasis, lipids participate in the entire cascade of tumorigenesis, metastasis, drug resistance, and immune evasion.^1^ By systematically reprogramming the synthesis, breakdown, transport, and storage of lipids, tumor cells construct a core mechanism that adapts to hostile microenvironments and maintains survival advantages.^2^

Within the intricate network of lipid metabolism, fatty acid (FA) metabolism occupies a central hub. As key precursors for phospholipids, glycerides, and signaling lipids,^3^ changes in FA metabolic flux directly influence the composition of these molecules, thereby regulating membrane integrity, fluidity, and lipid-mediated signal transduction. Under physiological conditions, FA synthesis (FAS), uptake, oxidation, and storage are tightly balanced to maintain cellular homeostasis. In contrast, tumor cells coordinately activate *de novo* FAS, enhance exogenous uptake, reprogram oxidative pathways, and remodel lipid droplet (LD) metabolism, thereby systematically reshaping FA metabolic homeostasis.^4^ This metabolic rewiring is not merely a consequence of malignant transformation but a key driver promoting tumor infiltration and relapse.

Hematological malignancies, which originate from hematopoietic and lymphoid lineages, are characterized by aggressive proliferative kinetics and heightened dependence on exogenous nutrients and biosynthetic precursors.^5^ Unlike solid tumors, these malignant cells reside in lipid-rich bone marrow and lymphoid microenvironments, engaging in active metabolic crosstalk with adipocytes, stromal cells, and immune cells.^6^ While metabolic reprogramming in these tumors commonly involves enhanced aerobic glycolysis and glutaminolysis, they also critically rely on the aberrant remodeling of lipid and FA metabolism to meet the demands of malignant proliferation.^7^ The reprogramming of FA metabolism in hematological malignancies exhibits pronounced disease-context dependency and microenvironment-driven features. Dominant activation of synthetic pathways in acute leukemia or enhanced uptake and storage of exogenous lipids in chronic lymphoproliferative disorders not only reflect differences in cellular origins and driver mutations, but also provide a rationale for precise interventions targeting metabolic weaknesses.^8^^,^^9^

Given the fundamental role of FA metabolic reprogramming in the biology of hematological malignancies, related genes and metabolites have emerged as clinically valuable prognostic markers.^10^ Moreover, targeting key nodes in this pathway represents a promising precision therapeutic strategy. Risk-scoring models based on FA metabolism-related gene expression profiles enable refined patient stratification, often outperforming traditional clinical staging systems. Concurrently, aberrant levels of specific lipid metabolites may serve as potential indicators of disease progression and prognosis.^11^ Interventions targeting FA metabolic vulnerabilities not only hold potential for directly eliminating tumor cells but may also synergize with conventional chemotherapy, targeted therapy, or immunotherapy to overcome drug resistance and improve outcomes.

Nevertheless, the field still lacks a systematic integrative framework to clarify the similarities and differences in metabolic dependencies across subtypes of hematological malignancies and to translate fundamental discoveries into biomarkers for precise stratification and clinically applicable combination regimens. This review aims to dissect the molecular mechanisms and functional consequences of FA metabolic reprogramming in hematological malignancies, integrating perspectives of metabolic vulnerability and disease heterogeneity to summarize subtype-specific metabolic dependencies. We further review current and emerging metabolic targeted interventions, focusing on combined treatment strategies that can overcome drug resistance and improve efficacy, to provide a new perspective for the development of precision therapy for environmentally specific metabolic dependence and provide theoretical support for improving the prognosis of patients with hematological malignancies.

## FA metabolism process

As an indispensable class of core lipid molecules in living organisms, the metabolic balance of FAs is crucial to maintain cell homeostasis. This section will focus on four key aspects of FA metabolism, including *de novo* synthesis, extracellular uptake, β-oxidation, and storage, systematically explaining their regulatory mechanisms and revealing how these processes work together to meet the energy supply and biosynthetic demands of cells.

### FA uptake

FA uptake is central to cellular maintenance of lipid homeostasis. Essential FAs, including polyunsaturated FAs, must be obtained from the diet of mammals, and their transmembrane transport efficiency directly determines the lipid composition and energy balance of cells.^12^

Cellular uptake of FAs relies on the coordinated action of specific transporters. CD36 functions as a high-affinity transmembrane transporter for long-chain FAs, facilitating their translocation across the plasma membrane lipid bilayer and thereby directly influencing cellular lipid homeostasis and energy metabolism.^13^

Fatty acid-binding proteins (FABPs) are important intracellular lipid chaperones responsible for FA transport and are highly expressed in metabolically active tissues. Based on their subcellular localization, they can be categorized into distinct functional subtypes. Plasma membrane-associated types primarily facilitate the transmembrane transport of FAs, whereas cytoplasmic types are responsible for regulating the intracellular distribution and targeted trafficking of FAs.^14^ These proteins maintain the stability of intracellular FA metabolism through complementary functions, and can form functional synergy with the transporter CD36 on the cell membrane to provide specific binding sites for the subsequent activation or nuclear transport of FAs.^15^

### FA synthesis

Under normal physiological conditions, the synthesis of FAs is mainly confined to professional adipogenic tissues, with most other cells relying on exogenous lipid uptake.^16^ Within these specialized tissues, FAS is an intracellular biochemical process catalyzed by multienzyme complexes to convert acetyl-CoA to long-chain saturated FAs for membrane synthesis, energy storage, and the generation of signaling molecules.

*De novo* FAS is initiated in the cytoplasm with acetyl-CoA as the core substrate. This central metabolite can be sourced through several biochemical routes. One major pathway involves the transport of mitochondrial citrate to the cytoplasm via solute carrier family 25 member 1 (SLC25A1), which is catalyzed by the rate-limiting enzyme ATP-citrate lyase (ACLY).^17^ In addition, acetate can be obtained by activation of acetyl-CoA synthetases (ACSS) or by the transfer of acetyl groups across organelles from mitochondria or peroxisomes.^18^ ACLY is a key node connecting glucose metabolism and lipid synthesis. When glucose utilization rate increases, mitochondrial citrate output increases, and cytosolic acetyl-CoA is catalyzed by ACLY, promoting the flow of carbon source from glycolysis to lipogenesis.

Acetyl-CoA in the cytoplasm is carboxylated by acetyl-CoA carboxylase (ACC) to produce malonyl-CoA. This reaction not only supplies the essential two-carbon units for FA elongation but also represents a critical regulatory point that helps balance FAS and oxidation.^19^ Malonyl-CoA is then condensed by fatty acid synthase (FASN) using acetyl-CoA as a primer to produce 16-carbon saturated palmitic acid.^20^ The initial product can be modified by sterol-CoA desaturase (SCD) to introduce unsaturated bonds or extend carbon chains, and eventually form a variety of FAs required for the synthesis of membrane phospholipids, cholesterol esters, and triglycerides.

### FA oxidation

Fatty acid oxidation (FAO) is the core pathway of bioenergy metabolism, which can efficiently decompose FAs to maintain cellular energy homeostasis under nutrient deprivation conditions. The essence of this process is the progressive breakdown of long-chain FAs to acetyl-CoA with the production of reduced NADH and FADH_2_ equivalents, which enter the mitochondrial respiratory chain to drive ATP synthesis.^21^

FAO mainly occurs in the mitochondrial matrix, while long-chain FAs cannot directly penetrate the mitochondrial inner membrane and rely on the carnitine shuttle system for transport. Fatty acylcarnitine is generated from long-chain fatty acyl-CoA catalyzed by carnitine palmitoyltransferase I (CPT1) at the outer mitochondrial membrane.^22^ There are tissue-specific isoforms of CPT1, and its activity is negatively regulated by malonyl-CoA, which is the rate-limiting step of FAO.^23^ Fatty acylcarnitine is then transported into the mitochondrial matrix by carnitine-acylcarnitine translocase (CACT), which is then reduced to fatty acyl-CoA by carnitine palmitoyltransferase II (CPT2), and enters the β-oxidation cycle.^22^ Acetyl-CoA produced by β-oxidation can enter the tricarboxylic acid cycle for further metabolism, while FAO can also produce NADPH through malic enzymes, to coordinate the needs of cellular biosynthesis and antioxidants, and realize the dual regulation of energy supply and redox balance.

### FA storage

In a physiological state, excessive FAs are catalyzed by diacylglycerol acyltransferases (DGATs) to form triglycerides, which are then mainly stored in white adipose tissue and skin as LDs.^24^ By maintaining a balance between lipid storage and breakdown, triglycerides regulate cellular lipid metabolism and reduce cell damage from lipid peroxidation.

Triglycerides stored in LDs are hydrolyzed by key enzymes such as hormone-sensitive lipase (HSL) and triglyceride lipase (ATGL) to release free FAs and glycerol when cellular energy demand increases or nutrients are scarce in the microenvironment.^25^ These released FAs enter the mitochondria for β-oxidation and are gradually decomposed into acetyl-CoA, which in turn efficiently produces ATP through the tricarboxylic acid and oxidative phosphorylation, providing the core energy support for cells to cope with metabolic stress.

## FA metabolism reprogramming in hematological malignancies

Lipid metabolism, functioning as a dynamic and interconnected pathway network, undergoes marked reprogramming across various cancers, with alterations in FA metabolism being particularly prominent. In hematological malignancies, this remodeling of FA metabolism is not a passive epiphenomenon but an actively established state of metabolic dependence. It enables malignant cells to support uncontrolled proliferation, adapt to microenvironmental stress, and ensure survival by dynamically regulating FA uptake, synthesis, storage, and oxidative decomposition. Table 1 delineates their dysregulation and consequent pathophysiological consequences.

**Table 1:** Expression changes of key enzymes of FA metabolism in hematological malignancies.

FA metabolic enzyme	Type of cancer	Expression	Function	Effect	Reference
CD36	Acute myeloid leukemia	Up-regulated	Uptake and transport FAs	Drive extramedullary migration and relapse of primitive cells, chemotherapy resistance	^95^^,^^173^
	Chronic myeloid leukemia	Up-regulated	Uptake and transport FAs	Imatinib chemotherapy resistance	^174^
	Chronic lymphocytic leukemia	Up-regulated	Uptake and transport FAs	Promote proliferation, improve the survival rate	^48^
	Mantle cell lymphoma	Up-regulated	Uptake and transport FAs	Bortezomib chemotherapy resistance	^63^
FABP4	Acute myeloid leukemia	Up-regulated	Transport FAs and enhance DNA methylation	Promote survival and proliferation	^175^
	Multiple myeloma	Up-regulated	Uptake FAs and regulate signal transduction	Promote proliferation and migration	^176^
FABP5	Acute myeloid leukemia	Up-regulated	Uptake and transport FAs	Promote proliferation, maintain triglyceride production	^143^
	Multiple myeloma	Up-regulated	Up-regulate MYC	Promote proliferation, invasion, and relapse, and infiltrate immune cells	^177^^,^^178^
ACLY	T-cell acute lymphoblastic leukemia	Up-regulated	Catalyze acetyl-CoA, ACLY–MYC feedforward loop	Promote the proliferation and transformation of thymic immature progenitor cells	^36^^;^^87^
	Acute myeloid leukemia	Up-regulated	Catalyze acetyl-CoA	Promote proliferation, inhibit chemotherapy resistance	^179^^,^^180^
	Acute lymphoblastic leukemia	Up-regulated	Catalyze acetyl-CoA, synthesize lipids, and β-oxidation	Promote the onset of disease	^181^
ACC1	Acute myeloid leukemia	Down-regulated	Catalyze acetyl-CoA to malonyl-CoA	Promote proliferation	^182^
	Multiple myeloma	Up-regulated	Catalyze acetyl-CoA to malonyl-CoA	Promote proliferation and transformation	^68^^,^^130^
ACC2	Acute myeloid leukemia	Down-regulated	Catalyze acetyl-CoA to malonyl-CoA	Activate FAO, promote proliferation	^183^
FASN	Acute myeloid leukemia	Up-regulated	Catalyze malonyl-CoA to palmitic acid, activate mTOR	Damage granulocyte differentiation, reduce autophagy, promote proliferation, and carfilzomib chemotherapy resistance	^184^^,^^185^
	Acute lymphoblastic leukemia	Up-regulated	Catalyze malonyl-CoA to palmitic acid	Dexamethasone chemotherapy resistance	^38^
	Diffuse large B cell lymphoma	Up-regulated	Catalyze malonyl-CoA to palmitic acid, induce PI3K–S6 kinase signaling, and regulate pERK/BCL-2 pathway	Promote oncogenic translation, proliferation, and invasion	^52^^,^^53^
	Cutaneous T cell lymphoma	Up-regulated	Catalyze malonyl-CoA to palmitic acid	Promote proliferation	^151^
	Mantle cell lymphoma	Up-regulated	Catalyze malonyl-CoA to palmitic acid	Promote proliferation and transformation	^87^
SCD1	Acute lymphoblastic leukemia	Up-regulated	Catalyze SFA to MUFA	Increase central nervous system burden	^37^
	Acute myeloid leukemia	Up-regulated	Catalyze SFA to MUFA	Promote proliferation and recurrence, reduce lipotoxicity, and prevent ferroptosis	^136^^,^^186^
	Chronic myeloid leukemia	Down-regulated	Catalyze SFA to MUFA	Promote leukemic stem cell proliferation, imatinib chemotherapy resistance	^187^
	Multiple myeloma	Up-regulated	Catalyze SFA to MUFA	Promote proliferation, reduce endoplasmic reticulum stress	^188^^,^^189^
FADS1	Acute myeloid leukemia	Up-regulated	Synthesize polyunsaturated FAs	Promote proliferation	^190^
ELOVL1	Acute lymphoblastic leukemia	Up-regulated	Extend long-chain FAs	Synthesize cell membranes, promote proliferation	^101^
ELOVL6	Acute myeloid leukemia	Up-regulated	Regulate PI3K–RAC signaling	Promote proliferation and transformation, regulate stemness	^191^
	Multiple myeloma	Down-regulated	Extend long-chain FAs	Reduce endoplasmic reticulum stress, bortezomib chemotherapy resistance	^192^
CPT1A	Acute myeloid leukemia	Up-regulated	Transport long-chain fatty acyl-CoA into mitochondria	Promote proliferation and recurrence, drive drug resistance	^146^^,^^148^
	Chronic lymphocytic leukemia	Up-regulated	Transport long-chain fatty acyl-CoA into mitochondria	Maintain the integrity of mitochondria, promote proliferation, and inhibit ibrutinib chemotherapy resistance	^149^^,^^193^
	Burkitt lymphoma	Up-regulated	Transport long-chain fatty acyl-CoA into mitochondria, promote FAO	Promote proliferation	^59^
	Acute lymphoblastic leukemia	Up-regulated	Transport long-chain fatty acyl-CoA into mitochondria	Promote proliferation	^194^
ACSL1	Chronic myeloid leukemia	Up-regulated	Regulate SIRT1/p53/p21	Promote cellular senescence	^195^
ACSL5	Acute myeloid leukemia	Up-regulated	Catalyze FA and acetyl-CoA to fatty acyl-CoA, and regulate Wnt/β-catenin signaling	Promote proliferation	^196^
LPL	Chronic lymphocytic leukemia	Up-regulated	Lipolysis	Promote proliferation, improve survival	^197^

Note: FA, fatty acid; CD36, cluster of differentiation 36; FABP, fatty acid-binding protein; ACLY, ATP citrate lyase; ACC, acetyl-CoA carboxylase; FASN, fatty acid synthase; SCD1, stearoyl-CoA desaturase 1; FADS1, fatty acid desaturase 1; ELOVL, elongase of very long chain fatty acids; CPT1A, carnitine palmitoyl-transferase 1A; ACSL, long-chain acyl-CoA synthases; LPL, lipoprotein lipase; SFA, saturated fatty acid; MUFA, monounsaturated fatty acid; FAO, fatty acid oxidation; mTOR, mammalian target of rapamycin; NAMPT, nicotinamide phosphoribosyltransferase.

Different hematological malignancies have evolved distinct FA metabolism-dependent patterns due to variations in driver genes and microenvironmental interactions, which form the basis for precision targeting. This metabolic heterogeneity, a comprehensive result of the interplay between genetic driver backgrounds, cellular origin characteristics, and unique tumor microenvironments (TMEs), highlights the need for subtype-specific analyses to identify actionable vulnerabilities.

### Heterogeneity in FA metabolic reprogramming across hematological malignancies

#### Leukemia

##### *Acute myeloid leukemia (AML)*

AML exhibits multifaceted reprogramming of FA metabolism, with a core therapeutic vulnerability likely residing in the deep dependency of leukemia stem cells (LSCs) on FAO.^26^ Residing within the hypoxic and nutrient-deprived bone marrow niche, LSCs preferentially utilize FAO over glycolysis to fuel their quiescent state, self-renewal capacity, and chemoresistance.^27^ This metabolic preference is reinforced through multiple mechanisms. On the one hand, hypoxia-inducible factors and other signaling pathways indirectly up-regulate carnitine palmitoyl-transferase 1A (CPT1A) expression, facilitating mitochondrial import and oxidation of long-chain FAs.^28^ On the other hand, FAO efficiently generates substantial ATP and NADPH, which are essential for maintaining redox homeostasis and scavenging reactive oxygen species to protect stem cell genomic integrity.^29^ Specific genetic lesions, including mutations in isocitrate dehydrogenase (IDH), nucleophosmin 1 (NPM1), and FMS-like tyrosine kinase 3-internal tandem duplications (FLT3-ITD), further entrench this metabolic state.^30^ Through mechanisms involving epigenetic reprogramming and transcriptional up-regulation of metabolic enzymes, these alterations cooperatively lock AML cells into an FAO-dependent metabolic program, thereby enhancing survival and contributing to therapy resistance. Notably, the metabolic heterogeneity of AML is rooted in the different survival requirements corresponding to its cellular hierarchy. As LSCs differentiate into rapidly proliferating blasts, their metabolic priorities shift from maintaining stemness and long-term persistence to supporting biosynthetic processes and rapid proliferation.^31^ Consequently, these blasts develop a high dependency on FAS to supply the phospholipids necessary for membrane biogenesis. This establishes a metabolic bifurcation wherein LSCs depend on FAO while proliferating blasts rely on FAS. Targeting these complementary pathways is therefore considered a promising strategy to eradicate distinct cellular subpopulations and prevent relapse. Notably, while normal hematopoietic stem cells possess basal FAO capacity, their metabolism remains significantly more plastic and less exclusively dependent on specific pathways under homeostasis compared with their malignant counterparts, which provides a theoretical basis for the above selective targeting.^32^

##### *Acute lymphoblastic leukemia (ALL)*

ALL is characterized by a profound metabolic reprogramming centered on FAS, which is driven by the extremely high proliferation rate of leukemic cells.^33^ Their rapid cell division creates a massive and sustained biosynthetic demand for lipid precursors, particularly membrane phospholipids, a demand that outstrips the supply capacity of relatively inefficient exogenous uptake pathways.^34^ This quantitative mismatch between supply and demand compels ALL cells to rely on autonomous and efficient endogenous FAS to sustain proliferation, forming a unique metabolic foundation for their survival. This reprogramming is not an isolated adaptation but is deeply integrated into the core oncogenic signaling networks driving the disease. Oncogenes systematically activate the entire metabolic pathway from acetyl-CoA production to long-chain FA synthesis by regulating transcription factors.^35^ In T-cell ALL, ACLY serves a dual role as a key initiating enzyme. Beyond providing acetyl-CoA substrate for lipid synthesis, ACLY influences histone acetylation to sustain the expression of oncogenes like MYC, thereby establishing a self-reinforcing feedforward loop that accelerates malignant progression.^36^ The metabolic dependency of ALL cells is further evidenced by their adaptation to specific tissue microenvironments. In B-cell ALL with central nervous system infiltration, cells must further rely on enzymes such as stearoyl CoA desaturase 1 (SCD1) to modify their self-synthesized FAs to maintain the functional integrity of the cell membrane in the face of the local lipid-deficient microenvironment.^37^ Importantly, this metabolic state, shaped by both intrinsic oncogenic drives and adaptive responses to external stress, creates a targetable therapeutic vulnerability. Pharmacological inhibition of this pathway can selectively induce death in drug-resistant leukemia cells and restore their sensitivity to conventional chemotherapy.^38^ In contrast, normal activated lymphocytes possess basal synthetic capacity but lack the same degree of absolute metabolic dependence, offering a potential therapeutic window for selective intervention. Thus, the dependence of ALL cells on FAS is a metabolic state driven by an intrinsic oncogenic program and solidified in response to external survival pressures.

##### *Chronic myeloid leukemia (CML)*

CML exhibits notable metabolic plasticity driven by the breakpoint cluster region-ABL proto-oncogene 1 (BCR-ABL1) oncoprotein, with a key vulnerability arising from the profound, therapy-induced dependency of LSCs on exogenous FA uptake and oxidation.^39^ To maintain quiescence and long-term survival, these stem cells rely heavily on FAO to meet their bioenergetic needs while avoiding the generation of detrimental levels of reactive oxygen species.^40^ When faced with selective stress such as tyrosine kinase inhibitor treatment or nutrient deprivation, cells synergistically down-regulate FAS and up-regulate the expression of FA transporter CD36 and oxidative rate-limiting enzyme CPT1A by activating stress response pathways such as Sirtuin1 (SIRT1)–AMP-activated protein kinase (AMPK).^9^^,^^41^ This reprogramming establishes an efficient uptake-oxidation circuit, enabling cells to extensively uptake and utilize exogenous FAs from the microenvironment. Through oxidative phosphorylation, this process generates substantial ATP, compensating for the disruption of canonical energy metabolism caused by treatment. This metabolic shift is not an intrinsic property of CML cells but a distinct survival strategy enacted under therapeutic selection, thereby constituting an acquired metabolic vulnerability. Furthermore, specialized niches such as the gonadal adipose tissue provide substantial support for this metabolic mode by supplying exogenous lipids, further entrenching the survival dependence of the stem cells on this pathway.^42^ Therefore, this metabolic state is established by intrinsic cellular requirements, powerfully amplified by therapeutic selection, and persistently supported by the microenvironment, and constitutes the irreplaceable survival dependence of CML leukemic stem cells. Since normal myeloid cells do not exhibit a comparably rigid dependence on this specific uptake–oxidation axis, its targeted disruption presents a promising strategy.^43^ This approach can selectively impair the maintenance and drug-resistant persistence of LSCs, offering a mechanistically grounded avenue to overcome therapeutic resistance in CML.

##### *Chronic lymphocytic leukemia (CLL)*

CLL has evolved a distinct metabolic adaptation, characterized by a profound reliance on the uptake, storage, and utilization of exogenous FAs from the lipid-rich lymphoid microenvironment. Unlike rapidly proliferating leukemias, CLL cells are usually in a state of cell cycle arrest or slow proliferation. Their metabolic core is not to support rapid biosynthesis, but to maintain their long-term survival and competition in lymphoid tissues. This metabolic dependency stems from an intrinsic deficit in biosynthetic capacity. In contrast to normal B cells, which can flexibly switch between uptake and synthesis, CLL cells have a serious lack of autonomous synthesis ability due to the low expression of FA synthase, thus forming a metabolic dependence on exogenous lipid uptake.^44^ To meet this ongoing demand, CLL cells actively up-regulate the expression of key molecules such as CD36 and lipoprotein lipase (LPL) through constitutively activated B-cell receptor (BCR) signaling.^45^ The latter hydrolyzes circulating lipoproteins like very-low-density lipoprotein, releasing free FAs for cellular uptake.^46^ Part of the FAs entering the cells enter the mitochondria for oxidation, providing basic energy for the resting cells.^47^ The other fraction is esterified and stored as triglycerides in large LDs.^48^ These LDs function not merely as inert energy reservoirs but as dynamic organelles that sequester excess lipids to prevent lipotoxicity and offer protection against oxidative stress. Thus, CLL cell survival is locked into a metabolic cycle of efficient uptake and dynamic storage. This cycle meets the unique needs of its long-term survival in the resting state and coping with microenvironmental fluctuations and competitive pressures. This metabolic dependence, shaped by both insufficient synthesis and enhanced uptake, constitutes a distinct therapeutic vulnerability of CLL.

#### Lymphoma

##### *Diffuse large B-cell lymphoma (DLBCL)*

The key metabolic vulnerability of DLBCL lies in the deep synergistic and self-reinforcing loops of *de novo* FAS and the core oncogenic signaling network.^49^ Whether driven by the constitutively active BCR/cluster of differentiation 40 (CD40)–phosphoinositide 3-kinase (PI3K)-protein kinase B (AKT)-mechanistic target of rapamycin (mTOR) axis in the germinal center B-cell-like subtype or by MYC overexpression, these signals converge on the transcription factor sterol regulatory element binding protein 1 (SREBP1).^50^^,^^51^ This convergence leads to the transcriptional up-regulation of the full suite of FAS genes, creating a dual-layered dependency on lipogenesis to meet the demand for membrane phospholipids required for MYC-driven hyperproliferation.^52^ During this reprogramming, FASN transcends its classical role as a metabolic enzyme, evolving into a pivotal signaling node. It catalyzes palmitic acid, a lipid precursor essential for the maintenance of PI3K-AKT-mTOR and BCL-2 pro-survival signals.^53^ Meanwhile, FASN can also stabilize oncoproteins such as c-Met through non-metabolic functions.^54^ This constructs a self-reinforcing vicious cycle of signal-driven synthesis and synthetic feedback maintenance signals, making cancer cells addictively dependent on them. It is this vicious cycle that makes DLBCL cells lose the metabolic plasticity of normal B cells and cannot switch between synthesis and uptake pathways flexibly, resulting in unique sensitivity to FASN inhibition. Inhibition of FASN not only disrupts membrane biosynthesis but also simultaneously disrupts key survival signaling networks, resulting in synergistic lethal effects.^55^^,^^56^ Clinical evidence shows that high expression of FASN is associated with poor prognosis of DLBCL, and inhibition of FASN can effectively induce cell apoptosis and enhance chemotherapy sensitivity.^57^

##### *Burkitt lymphoma*

Metabolic reprogramming in Burkitt lymphoma is characterized by the synergistic activation of *de novo* FAS and oxidation pathways. This represents a highly specialized, MYC-driven adaptation designed to meet the extraordinary biosynthetic demands of one of the most rapidly proliferating human cancers. Faced with immense anabolic pressure, MYC directly transactivates key genes such as FASN, powerfully driving *de novo* lipogenesis to supply the essential precursors for rapid membrane expansion.^58^ Concurrently, to match this extremely high biosynthetic energy consumption and avoid the metabolic stress caused by excessive dependence on glycolysis, Burkitt lymphoma cells synergistically maintain efficient FAO capacity.^59^ This indicates that under the dominance of MYC, Burkitt lymphoma metabolism presents a highly specialized pattern with synthesis as the absolute core and oxidation as the energy guarantee. This high-intensity synergy is the root of the metabolic vulnerability of Burkitt lymphoma. Normal B cells have a low demand for proliferation and do not require simultaneous activation of two energy-consuming pathways. This makes targeting any link in this coordinated metabolic system a promising therapeutic strategy.

##### *Mantle cell lymphoma (MCL)*

MCL exhibits a distinct pattern of FA metabolic reprogramming, characterized by the coordinated rewiring of both FAS and exogenous FA uptake pathways. This reprogramming not only represents pathway activation but also forms the dependence of tumor cells on specific lipid pathways for survival under the joint action of oncogenic drive and microenvironmental stress, constituting an exploitable metabolic vulnerability. MCL cells often present with concurrent MYC activation and Cyclin D1 overexpression,^60^ which not only drive cell cycle progression but also upregulate FASN expression via the activation of the SREBP transcription factor, thereby supporting continuous membrane biogenesis.^61^ Inhibition of this synthetic pathway directly induces apoptosis, confirming its indispensability for maintaining core proliferative capacity.^62^ However, MCL cells are often localized in the bone marrow microenvironment with fluctuating nutrition *in vivo* and face therapeutic pressures such as proteasome inhibitors. To this end, MCL cells activate CD36-mediated exogenous FA uptake to generate energy through FAO, helping cells resist bortezomib drug-induced stress.^63^ Notably, MCL has evolved a unique alternative metabolic pathway to cope with microenvironmental challenges. These cells often exhibit high expression of ACSS1, an enzyme that converts microenvironmental acetate into acetyl-CoA.^64^ This mechanism helps maintain tricarboxylic acid cycle flux and biosynthetic capacity under glucose deprivation, constituting a crucial metabolic backup system. Normal lymphocytes have neither such a strong need for continuous synthesis nor the full set of alternative pathways to cope with extreme stress. Therefore, combined targeting of its intrinsic synthesis-dependent and inducible uptake and backup pathways is expected to synergistically disrupt this multi-level metabolic adaptation network and provide a new potential strategy for overcoming treatment resistance.

#### Plasma cell disorders

##### *Multiple myeloma (MM)*

The essence of metabolic reprogramming of MM is an adaptive process driven by the bone marrow microenvironment, with exogenous lipid dependence as the core. MM cells remodel lipid uptake and storage pathways to tightly couple their survival and proliferation to the extracellular lipid pool supplied by adipocytes. This non-autonomous metabolic dependence constitutes its unique therapeutic vulnerability. The bone marrow cavity colonized by MM cells is rich in adipocytes,^65^ providing them with a unique metabolic niche. MM cells secrete cytokines to induce lipolysis of adjacent adipocytes, and the released free FAs are efficiently taken up by MM cells through transporters such as FABPs and CD36.^66^^,^^67^ Unlike tumors with active energy metabolism, FAs entering MM cells are mainly esterified and stored in LDs.^68^ These LDs not only meet biosynthetic requirements but also form physical resistance by sequestering chemotherapeutic drugs.^69^ The lipid richness of the bone marrow TME and stromal cell signaling together drive this reprogramming. Adipocytes provide a continuous source of FA, and stromal cells enhance lipid uptake and storage in MM cells through the PI3K–AKT–mTOR pathway, forming a microenvironment-dependent metabolic cycle.^70^ Normal plasma cells have a short life span and do not need to establish such a complex and continuous extracellular lipid supply chain. The long-term survival and expansion of MM cells in the bone marrow form a functional dependence on exogenous lipid supply and LD storage. Therefore, interfering with lipolysis supply of adipocytes, blocking CD36 or FABP-mediated uptake, inhibiting LD formation or LD homeostasis, and interfering with stromal cell signaling input are expected to weaken this dependent circuit at both the source and intracellular end, thereby improving chemosensitivity and reducing the occurrence of drug resistance.

## The factors modulating FA metabolism reprogramming in hematological malignancies

FA metabolism reprogramming in hematological malignancies represents a highly ordered adaptive program rather than a stochastic metabolic alteration. Different malignancies rely predominantly on distinct facets of FA metabolism, a preference dictated by their intrinsic proliferative rate, cell state, and unique microenvironment. This metabolic reprogramming is initiated by disease-specific oncogenic drivers that fundamentally reshape the metabolic direction of cellular processing of FAs. Subsequently, specialized transcriptional programs and signaling networks systematically constrain cellular metabolic plasticity while stabilizing dominant pathways. Persistent microenvironmental pressures, including hypoxia, nutrient limitation, and lipid availability, further consolidate these programs by selectively favoring cells committed to the most compatible FA metabolic mode. Together, these processes transform FA metabolic plasticity into rigid, disease-specific metabolic addictions that constitute predictable and targetable vulnerabilities.

### Oncogenic drivers define metabolic direction

Distinct oncogenic lesions establish the primary metabolic orientation of malignant cells by aligning FA metabolism with the core biological needs of each disease subtype, such as rapid proliferation, long-term quiescence, or stress resistance.

#### *MYC-driven anabolic pressure*

In highly proliferative lymphoid tumors such as DLBCL, Burkitt lymphoma, and MCL, MYC acts as a core oncogenic transcription factor that highly couples cell cycle acceleration to the need for membrane lipid synthesis.^52^^,^^71^^,^^72^ MYC directly activates promoters such as FASN, ACC, SCD, and collaborates with SREBP1 to continuously promote high-intensity *de novo* FAS to maintain the demand for explosive membrane biosynthesis.^73^ Under MYC-driven synthetic pressure, exogenous lipid uptake is insufficient to fully meet the proliferative demand, shifting FAS from an alternative metabolic pathway to an irreplaceable survival pillar. With disease progression, these tumors gradually increase their dependence on the FAS pathway, exhibiting synthesis-dominated and uptake-assisted metabolic plasticity. In contrast, in plasma cell neoplasms such as MM, MYC-associated metabolic programs are preferentially oriented toward sustaining lipid availability and redox homeostasis to buffer secretory stress, rather than primarily driving proliferative growth.^74^

#### *TP53 status determines metabolic plasticity*

In hematological malignancies, the reprogramming of FA metabolism is closely related to the status of tumor protein 53 (TP53), and its regulatory pattern shows unique differences from that of solid tumors. In the presence of intact TP53 function, cells are able to dynamically adjust between FAS and oxidation in response to energy or oxidative stress.^75^ This property is particularly evident in indolent tumors such as CLL, making them dependent on FAO for long-term survival without a complete switch to synthetic dependence.^76^ In contrast, the loss of metabolic plasticity mediated by TP53 mutations directly promotes the remodeling of FA metabolism toward synthesis. Loss of TP53 function not only impairs AMPK-mediated metabolic compensation but also directly or indirectly up-regulates the expression of FASN and SCD1, forcing cells to tilt toward a synthetically dominant mode. This shift is particularly prominent in AML and aggressive lymphomas with TP53 mutations, making FAS a difficult metabolic bottleneck to bypass.^77^^,^^78^

#### *Spectrum-specific oncogenes*

Partial oncogenic events can impose highly focused metabolic constraints in a given disease. For example, the FLT3-ITD mutation in AML increases both FAS and desaturation requirements and sensitivity to oxidative stress and ferroptosis,^31^^,^^79^ thus locking cells into a dual dependence on the FASN–SCD axis. This pathway dependence is absent in normal hematopoietic progenitor cells and constitutes a clear therapeutic window.

### Transcriptional programs establish long-term FA metabolic identity

Transcription factors determine the long-term orientation of lipid metabolism in different hematological malignancies by integrating genes involved in FA metabolism into the core transcriptional program of the cell. In contrast to transient metabolic stress responses, these transcriptional programs enable tumor cells to maintain a stable pattern of FA utilization under different environmental conditions.

#### *SREBP*

SREBP functions as a transcriptional execution hub that converts oncogenic and environmental signals into sustained FA-generating outputs. Activation of SREBP in a variety of highly proliferative hematological malignancies makes *de novo* FAS an irreplaceable survival requirement by maintaining a constant supply of lipids required for membrane biogenesis.^80^ In central nervous system leukemia, sustained SREBP activity enables autonomous lipid generation under lipid-limited conditions, thereby reducing dependence on exogenous lipid utilization.^81^ In contrast, in diseases characterized by high immunoglobulin synthesis, such as MM, SREBP-driven FAS serves more for the maintenance of endoplasmic reticulum (ER) homeostasis and LD formation than simply supporting cell proliferation.^82^

#### *PPAR*

The peroxisome proliferator-activated receptor (PPAR) family plays a complementary role in the refinement of FA metabolic identity. PPARα can maintain energy homeostasis in a long-term resting state in low-proliferative tumors such as CLL by activating the FA uptake and oxidation program.^83^ PPARγ is more likely to promote FA uptake and storage, reinforcing the dependence on exogenous FAs in diseases that are highly coupled to a lipid-rich microenvironment.^84^ In addition to the dominant role of PPARα and PPARγ, PPARδ may also regulate FAO and mitochondrial fitness in specific contexts,^85^ but current evidence suggests that PPARδ affects metabolic capacity more than defining a dominant FA metabolic identity. Through the synergistic action of these transcriptional regulatory programs, different diseases gradually form a stable and differentiated FA metabolic orientation.

### Signaling pathways dynamically reinforce and constrain FA metabolic choices

After the direction of FA metabolism has been established by the transcriptional program, the signaling pathway senses growth signals, energy status, and microenvironmental stress to dynamically strengthen the established metabolic program, while inhibiting alternative pathways, thereby gradually reducing the metabolic selection space.

#### *PI3K–AKT–mTOR*

The PI3K–AKT–mTOR pathway constitutes the core hub of proanabolic signaling, which is continuously activated in a variety of hematological malignancies and acts synergistically with other oncogenic signaling pathways to enhance and stabilize the reprogramming of FA metabolism.^86^ During this process, sustained AKT activation directly bridges growth-promoting signals to metabolic regulation, providing a steady driving force for downstream FAS programs.^87^ With the support of AKT signaling, mechanistic target of rapamycin complex 1 (mTORC1) up-regulates the expression of key gene programs for FAS by promoting the maturation and nuclear translocation of SREBP,^88^ thus synchronizing FAS with hematological malignancies' cell growth and cell cycle progression, thereby enhancing the dependence on the FAS pathway. At the same time, mTORC2 enables long-term maintenance and greater stability of programs related to FAS by maintaining the persistence of AKT signaling and supporting metabolic adaptation of cells under stress conditions.^89^ In hematological malignancies, the PI3K–AKT–mTOR pathway drives fundamental metabolic remodeling through the synergy of its complex mTORC1 and mTORC2. This pathway not only enhances metabolic flux but also establishes programs such as FAS as a core metabolic state, thereby switching plastic metabolic adaptations to rigid metabolic dependencies and thus creating targetable therapeutic vulnerabilities.

#### *AMPK*

AMPK acts as a central sensor of cellular energy status and is activated under metabolic stress conditions such as glucose deprivation or hypoxia.^90^ Its primary function in this context is to suppress FAS while preserving FAO capacity, thereby providing an efficient and sustainable energy supply. In LSC populations of AML and CML, AMPK-mediated metabolic regulation supports cellular quiescence and long-term survival, enabling cells to maintain energy homeostasis under unfavorable metabolic conditions.^41^^,^^91^ Importantly, AMPK does not establish a new FA metabolic program in these settings; rather, it functions as a stress-responsive reserve pathway that preserves limited metabolic adaptability within an otherwise fixed metabolic framework, thereby supporting tumor cell survival.

#### *Lineage- and context-specific signaling pathways*

In addition to core metabolic regulators, several lineage- and context-specific signaling pathways, including Janus kinase (JAK)–signal transducer and activator of transcription (STAT), BCR–ABL, and NOTCH, further refine metabolic dependency patterns in different diseases by integrating cytokine or lineage-specific signaling to transform external niche stress into sustained stimulation of specific FA metabolic pathways.^86^^,^^92^ Rather than independently determining FA metabolic directionality, these pathways reinforce and sustain pre-established metabolic programs by promoting lipid uptake, utilization, and survival signaling in a disease- and context-dependent manner.^93^ In myeloid malignancies such as CML, constitutive BCR–ABL signaling provides continuous metabolic support that converges on downstream lipid metabolic pathways,^9^^,^^94^ whereas cytokine-driven JAK–STAT activation in lymphoid and myeloid neoplasms facilitates long-term survival by enhancing access to microenvironment-derived FAs.^95^^,^^96^ Similarly, NOTCH signaling integrates developmental and lineage cues to modulate lipid metabolism under specific cellular states.^97^ Despite their molecular diversity, these pathways converge functionally to stabilize disease-specific FA metabolic dependencies, ensuring that metabolic programs selected by oncogenic and transcriptional drivers are maintained over time.

### Microenvironmental pressures stabilize FA metabolic addiction

The TME is not the initial event of FA metabolic reprogramming, but by continuously applying selective pressures such as hypoxia, acidification, and nutrient competition, the reversible metabolic adaptation established by oncogenes is transformed into stable metabolic dependence, thereby locking disease-specific FA metabolic programs in the bone marrow and lymphoid niches.

#### *Hypoxic environment*

Under hypoxic stress, mitochondrial oxidative phosphorylation is limited, which aggravates the imbalance between energy and redox of cells, so that they cannot continuously rely on FAO for energy. In the long-term structural hypoxia microenvironment, such as bone marrow, leukemia and lymphoma cells therefore turn to enhanced FA uptake and lipid management to maintain membrane homeostasis, biosynthetic demand, and redox balance.^58^^,^^98^ Meanwhile, hypoxia further solidifies the dependence on specific FA metabolic pathways by regulating the degree of lipid desaturation and membrane composition,^99^ thereby stabilizing the disease-specific lipid metabolism pattern under oxygen limitation.

#### *Acidosis*

The acidotic state in the TME further shapes FA metabolic dependence through lactate accumulation and decreased extracellular pH. In highly proliferative or biosynthetically active hematological malignancies, the acidic environment can enhance the protonation state of FAs and promote their passive transmembrane transport, while driving lipid storage and LD formation, thereby providing energy and lipid buffer pool for tumor cells.^9^ This metabolic strategy centered on lipid uptake and storage helps to maintain membrane stability and redox homeostasis, and improves cellular tolerance to metabolic and therapeutic stresses.^100^ Through sustained selective pressure, acidosis acts in concert with hypoxia to further stabilize cell populations dependent on specific FA metabolic pathways.

## Roles of FA metabolism reprogramming in hematological malignancies

FA metabolism reprogramming exerts pleiotropic and context-dependent effects on the biology of hematological malignancies. Rather than serving as a passive metabolic adaptation, rewired FA utilization actively sustains malignant survival, reinforces oncogenic signaling, constrains cell fate decisions, and promotes resistance to therapeutic stress, as shown in Figure 1. Importantly, the functional consequences of FA metabolic reprogramming are tightly coupled to disease-specific metabolic dependencies established by oncogenic drivers and microenvironmental pressures.

**Figure 1:**
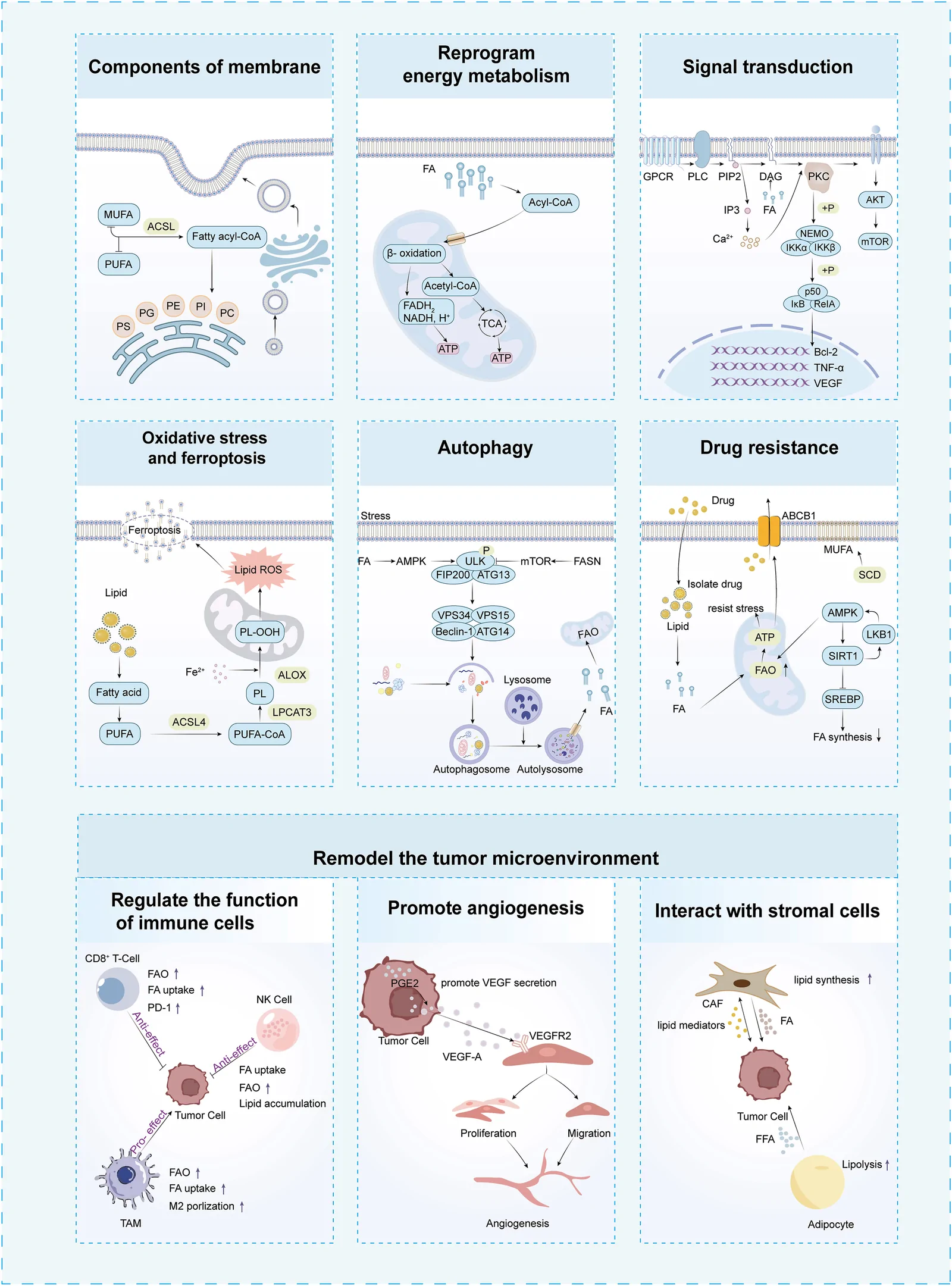
Biological functions of fatty acid metabolism. Fatty acid metabolism in hematological malignancies facilitates tumor cell proliferation by enhancing the synthesis of cellular membranes and modulating energy metabolism. It influences cell signaling through phosphorylation of diacylglycerol (DAG) and pathways like NF-κB and AKT–mTOR. Fatty acid metabolism regulates several essential biological processes within cells, including apoptosis and oxidative stress, potentially contributing to ferroptosis, and enhances drug resistance and remodels the tumor microenvironment.

### Sustaining bioenergetic and biosynthetic demands

FA metabolism provides essential bioenergetic and biosynthetic support for malignant cell survival and expansion in hematological malignancies. In highly proliferative hematological malignancies, such as ALL and aggressive B-cell lymphomas, FAS represents a critical metabolic foundation for rapid cell division.^101^^,^^102^ Continuous lipid synthesis supplies phospholipid precursors required for membrane biogenesis, thereby sustaining cell cycle progression and clonal expansion.^103^ Because the demand for membrane phospholipids is tightly coupled to proliferative rate, malignant cells require a continuous lipid supply that is precisely coordinated with cell cycle progression, but the fluctuating extracellular lipid supply is difficult to meet this demand.^104^ Therefore, such tumor cells gradually form a metabolic model dominated by synthesis, placing the FAS pathway at the core of their survival and proliferation. In this context, inhibition of lipogenic pathways directly deprives malignant cells of the material basis required for growth, leading to marked impairment of proliferative capacity and viability.

By contrast, in quiescent or therapy-stressed cell populations, including stem and progenitor compartments in AML and CML, FAO serves as a highly efficient source of ATP while simultaneously supporting NADPH production through associated metabolic pathways.^39^^,^^105^ This coupling of energy generation and redox homeostasis is particularly advantageous under conditions of glucose limitation or therapeutic stress, where reliance on the relatively inefficient glycolytic pathway alone is insufficient to sustain cellular survival.^106^ By aligning FA utilization with disease-specific cellular states such as proliferation, quiescence, or stress adaptation, FA metabolism establishes bioenergetic programs that are difficult to compensate through alternative nutrient pathways, thereby creating metabolic dependencies linked to cellular state.

### Remodeling membrane architecture and signal transduction

Beyond serving as metabolic substrates, FAs are critical structural and signaling components that shape membrane organization and signal transduction in malignant cells. Alterations in FAS, desaturation, and utilization reshape signaling output at multiple levels by remodeling membrane architecture, disrupting the homeostasis of lipid-derived second messengers, and engaging key FA metabolic enzymes in the regulation of signaling networks.

FA metabolism-driven changes in lipid composition influence membrane fluidity, lipid raft organization, and receptor spatial distribution.^107^ In aggressive lymphoid malignancies, enhanced FAS and desaturation promote membrane environments that favor sustained activation of growth and survival signaling pathways.^31^^,^^49^ By maintaining appropriate membrane fluidity, FA metabolism facilitates clustering of surface receptors and signaling complexes, thereby amplifying signaling output from pathways such as PI3K–AKT–mTOR and BCR-driven cascades.^108^ In lipid-restricted microenvironments, reliance on intrinsic FA metabolic programs further supports maintenance of signaling competence under limited extracellular lipid availability.^109^

Concurrently, aberrant FA metabolic flux alters the generation and interconversion of lipid second messengers, modulating the activity of multiple downstream effector proteins and kinases. Tumor cell dependence on specific FA metabolic pathways is partly driven by the intrinsic requirement to sustain lipid metabolite-driven oncogenic signaling networks, enabling FA metabolism to directly regulate signaling strength, duration, and feedback control.^110^ In addition, under quiescent or stress-adapted states, key FA metabolic enzymes influence signaling balance by regulating metabolite availability.^53^ Collectively, through coordinated structural remodeling and signaling regulation, FA metabolism tightly couples metabolic state to cell fate determination.

### Shaping redox balance, cell death susceptibility, and stress tolerance

The functional coupling between FA metabolism, oxidative stress, and programmed cell death defines a critical axis of metabolic dependency in hematological malignancies. By regulating NADPH production, lipid peroxidation, and mitochondrial function, FA metabolic programs directly shape the extent of oxidative damage and the threshold for cell death under unfavorable conditions.

Enhanced FAO supports redox homeostasis by sustaining mitochondrial respiration and promoting NADPH generation, thereby buffering reactive oxygen species produced during metabolic or therapeutic stress. This function is particularly relevant in stem-like or quiescent tumor cell populations, which rely on FAO-mediated redox control to support long-term survival under glucose restriction or hypoxia.^111^^,^^112^ In contrast, alterations in FAS and desaturation remodel membrane lipid composition and markedly influence susceptibility to lipid peroxidation. Ferroptosis is a form of programmed cell death driven by iron-dependent accumulation of lipid peroxides,^113^ and the abundance of polyunsaturated FAs in cellular membranes is a key determinant of sensitivity.^114^ Owing to their strong dependence on specific FA metabolic pathways, malignant cells must precisely maintain the balance between lipid saturation and antioxidant capacity, and disruption of this balance readily predisposes them to ferroptotic cell death.^115^, ^116^, ^117^

In addition, FA metabolic reprogramming regulates autophagy through metabolic sensing signaling nodes such as mTOR and AMPK. Under stress conditions, changes in lipid utilization and cellular energy status converge on these pathways, resulting in suppression of mTOR activity and activation of AMPK, thereby promoting autophagy as an adaptive survival response.^118^^,^^119^ Through this signaling-mediated mechanism, autophagy enables malignant cells to maintain metabolic homeostasis and tolerate prolonged nutrient limitation or therapeutic stress.^120^ Importantly, this stress-adaptive state remains dependent on intact FA metabolic support, thereby converting a survival advantage into a therapeutically exploitable metabolic vulnerability.

### Driving therapeutic resistance and disease persistence

In hematological malignancies, FA metabolic reprogramming represents a crucial adaptive mechanism by which tumor cells acquire resistance to chemotherapy. This adaptation manifests as differential reliance on specific FA metabolic pathways across disease types. In myeloid leukemias, enhanced FAO supports tumor cell survival under chemotherapy- or targeted therapy-induced stress by maintaining mitochondrial function and redox balance.^41^^,^^121^ In contrast, in lymphoid malignancies, sustained FAS helps preserve membrane integrity and signaling competence, thereby attenuating the cytotoxic effects of antiproliferative therapies.^57^ The TME further amplifies this resistant phenotype. Bone marrow adipocytes can supply exogenous FAs to support the survival of tumors such as MM, while the formation of intracellular LDs can physically sequester chemotherapeutic agents, reducing their effective intracellular exposure.^69^

### Remodeling the TME

FA metabolic reprogramming extends beyond malignant cells to remodel the TME, thereby further stabilizing disease-specific metabolic dependencies in hematological malignancies. By regulating local FA availability and metabolic interactions within tissue niches, dysregulated FA metabolism progressively shapes a microenvironment characterized by immune suppression, vascular adaptation, and stromal metabolic support.

Aberrant FA metabolism contributes to the establishment of an immunosuppressive microenvironment. By increasing lipid supply and enhancing FA utilization efficiency, malignant cells induce lipid accumulation and metabolic exhaustion in cytotoxic T cells and natural killer cells, thereby impairing anti-tumor immune effector function.^122^^,^^123^ In lipid-enriched niches, tumor cells maintain immune evasion through FA-driven metabolic competition, gaining a selective survival advantage.^124^

Altered FA metabolism also participates in the regulation of tumor-associated angiogenesis. Lipid-derived metabolites and FA-dependent signaling activities promote endothelial cell activation and vascular remodeling,^125^ improving nutrient and oxygen delivery under conditions of tumor expansion or metabolic stress. This vascular adaptation further consolidates FA-dependent growth programs.^126^

In addition, metabolic coupling between malignant cells and stromal components constitutes a fundamental basis of microenvironmental remodeling. Within the bone marrow niche, stromal cells and adipocytes supply exogenous FAs that are taken up and utilized by tumor cells to support mitochondrial function, redox homeostasis, and stress tolerance.^127^ This metabolic symbiosis is particularly evident in adipocyte-rich microenvironments such as those found in MM, where stromal lipid support progressively reduces metabolic plasticity and reinforces dependence on specific FA metabolic pathways.^128^

## Therapeutic targets

Reprogramming of FA metabolism provides a unique therapeutic target for hematological malignancies. At present, small molecule inhibitors targeting the key links of the whole chain of FA metabolism and its upstream regulatory hub have become research and development hotspots in the field of translational medicine. Their combination with chemotherapy and immunotherapy can improve the efficacy and reduce the risk of drug resistance through the synergistic mechanism of metabolic blockade plus killing/immune activation, as shown in Figure 2. It is worth noting that there is significant heterogeneity in the dependence of different disease subtypes on specific metabolic pathways, which is the core premise and theoretical basis for individualized targeted therapy.

**Figure 2:**
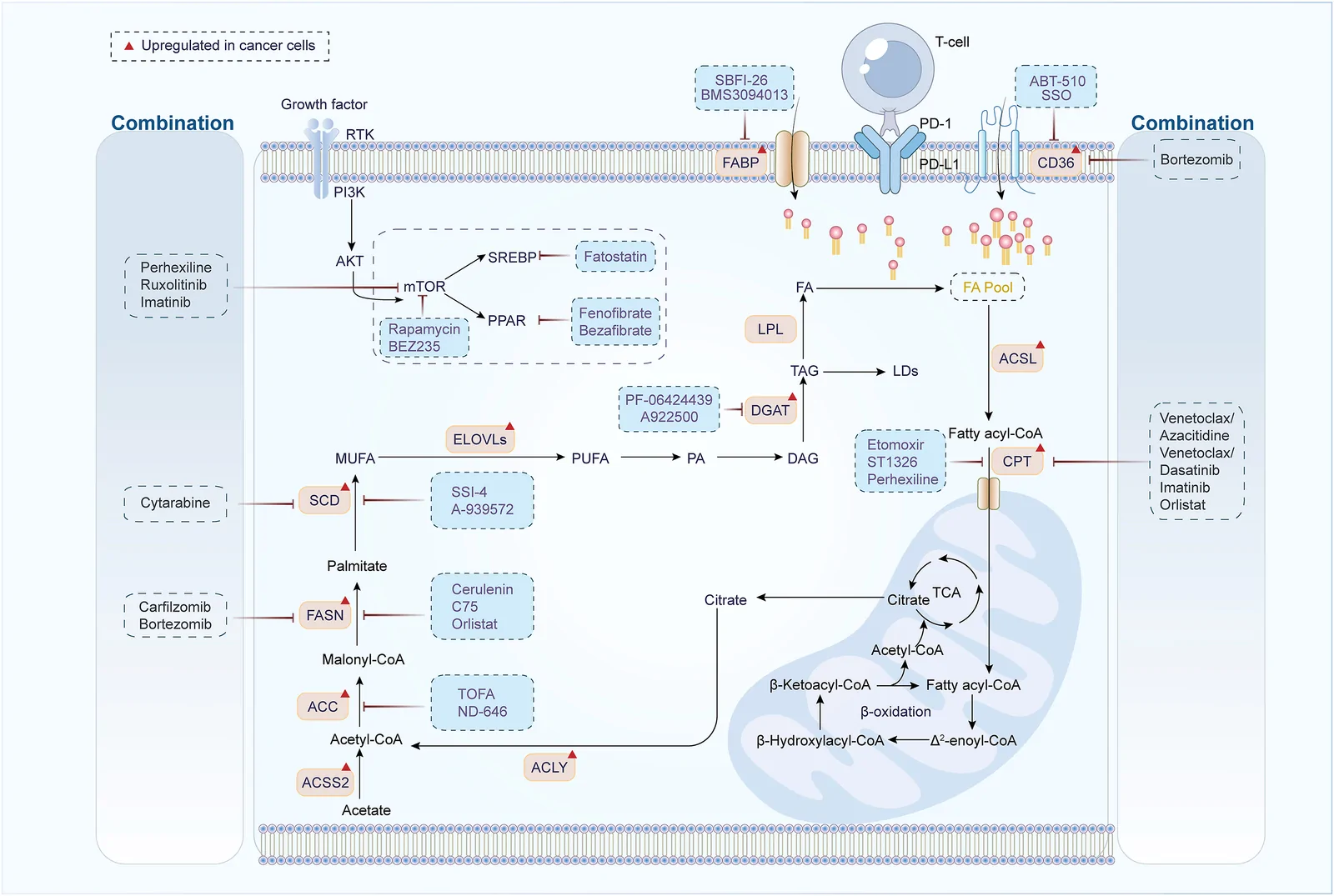
Therapeutic interventions on fatty acid (FA) metabolic reprogramming in hematological malignancies. The figure shows that blocking FA metabolic enzymes can interfere with FA uptake, synthesis, oxidation, and storage, providing therapeutic benefits. Combination therapy demonstrates the potential of multiple approaches to targeted FA metabolism. Key components are labeled for clarity. Molecular inhibitors are indicated in blue; the combination therapy segment is presented without background color. The arrows denote directional flow within metabolic pathways, and “T” symbols signify potential sites for targeted therapies.

### Core targets of disease-specific metabolic dependence

Guided by the pronounced heterogeneity of FA metabolic dependence across hematological malignancies, we organize actionable targets by the dominant dependency mode. Accordingly, targets are grouped into synthetic, uptake/storage, and oxidative dependencies.

#### Targeting synthetic dependence: Application of FASN, ACC, and SCD inhibitors in lymphoproliferative disorders

A variety of lymphoproliferative diseases, including ALL, DLBCL, and MCL, show dependence on the *de novo* synthesis of FAs and their subsequent modification processes. The addiction of FAS is driven by the oncogene signaling pathway, which provides membrane phospholipids, signaling lipids, and FAs required for cell membrane construction for the rapid proliferation of tumor cells. Interventions targeting the initiation, extension, and desaturation of the FAS pathway can directly destroy the material basis of tumor cell proliferation and then exert anti-tumor effects.

##### *ACC*

Targeting the upstream rate-limiting enzymes ACC and FASN is an important intervention strategy. ACC mediates the initial step of FAS. The early ACC inhibitor 5-tetradecyloxy-2-furoic acid (TOFA) can allosterically inhibit ACC activity, thereby effectively blocking the FAS pathway and inducing dose-dependent tumor cell death. In MYC, RAS, and BCR–ABL-driven malignant lymphoma models, TOFA could significantly induce tumor proliferation.^73^ TOFA also inhibited cell proliferation, and this effect could be reversed by exogenous FA supplementation in acute leukemia, confirming that its mechanism of action was to cut off the endogenous synthesis pathway of FAs.^129^ The synthetic small molecule ACC inhibitor ND-646 also demonstrated significant potential in inhibiting adipogenesis and hindering tumor cell growth in the preclinical model of MM.^130^

##### *FASN*

FASN is a core multifunctional enzyme that catalyzes FA chain elongation. Inhibition of FASN can interfere with the signaling network and biological processes required for tumor cell proliferation and stress response. Its natural inhibitor, cerulenin, can inhibit proliferation and trigger apoptosis in non-Hodgkin lymphoma, which is a potential candidate drug for the treatment of leukemia, but it lacks selectivity.^131^ The first synthetic FAS inhibitor, C75, is chemically stable. Preclinical studies show that it can induce apoptosis of DLBCL cell lines and show significant anti-tumor activity in primary exudative lymphoma cell lines.^54^^,^^132^ The approved FASN inhibitor Orlistat can effectively kill MCL cells by inducing cell death and stabilizing pro-apoptotic proteins.^133^ However, these first-generation inhibitors may be associated with substantial systemic metabolic side effects, including weight loss.^134^ TVB-2640, a new generation of FASN inhibitor, shows stronger anti-tumor potency,^135^ higher selectivity, and lower toxicity in solid tumors, suggesting that it has a good application prospect in hematological malignancies.

##### *SCD*

As a downstream key enzyme in the synthetic pathway, SCD is responsible for the conversion of SFAs to MUFAs. At present, a variety of SCD1 inhibitors are in the preclinical stage of cancer research. Among them, the novel SCD1 inhibitor SSI can amplify DNA damage induced by chemotherapeutic drugs, and SSI-4 shows significant anti-tumor activity in preclinical animal models of AML without significant side effects.^136^ The second-generation inhibitor A-939572 not only effectively induces apoptosis of AML cells, but also has a synergistic effect with the chemotherapy drug cytarabine.^137^ Tumor cells can compensate for the metabolic imbalance caused by SCD1 loss by up-regulating the expression of other desaturases, such as fatty acid desaturase 1/2 (FADS1/2),^138^ which suggests that future studies need to focus on the development of combined inhibition strategies for multiple desaturase nodes.

#### Targeting uptake and storage dependencies: The therapeutic potential of CD36, FABP, and DGAT inhibitors in MM and CLL

Hematological malignancy cells, such as MM and CLL, can interact with the lipid-rich bone marrow microenvironment and then show a high dependence on exogenous FA uptake and intracellular LD storage. This metabolic adaptation can help tumor cells cope with nutrient deprivation stress and enhance their survival advantage.

##### *CD36*

As a key receptor mediating the transmembrane uptake of long-chain FAs, the regulation of CD36 function has become the core direction of targeting FA uptake pathways. Thrombospondin-1 (TSP-1) is a known ligand of CD36, and its down-regulation is closely related to the progression of human hematological malignancies, including various lymphomas and leukemia. The combination of TSP-1 and CD36 can induce the apoptosis of megakaryoblastic leukemia cells, which may provide a new direction for the treatment of this type of leukemia.^139^ ABT-510, a mimetic peptide developed based on the structure of TSP-1, has entered phase Ⅱ clinical trials for relapsed/refractory lymphoma.^140^ The other targeted drug, sulfosuccinimidyl oleate (SSO), blocks CD36 function by forming irreversible binding to its FA binding site. The compound significantly reduced the oxygen consumption rate and induced dose-dependent apoptosis in an *in vitro* CLL cell model co-cultured with FAs.^141^ In addition, CD36 can be used as a marker of tumor cell subsets with metastatic initiation potential. SSO can also reduce the epithelial–mesenchymal transition phenotype and migration ability of palmitic acid-treated cells by targeting CD36.^142^ Although the above targeting strategies have shown good application potential, the efficacy and safety of SSO in clinical application are still controversial.

##### *FABP*

FABP family members are responsible for transporting the intracellular uptake of FAs to specific organelles. The inhibitors SBFI-26 and BMS309403 could disrupt the cell cycle progression of MM cells and interfere with the conduction of the MYC signaling pathway.^74^ Natural products Lycoris radiata alkali inhibit PPARγ and FABP5 expression level, through induction of apoptosis in the AML, as well as reduce the generation of triglycerides.^143^ The above studies suggest that simultaneous targeting of CD36 and FABP can synergistically block the transmembrane uptake and intracellular metabolism of FAs, thereby enhancing the anti-tumor effect of targeted therapy.

##### *DGATs*

For FA storage, DGATs act as the rate-limiting enzymes in the final step of triglyceride synthesis, and DGAT1 is highly expressed in MM.^65^ Its inhibitor A922500 has shown potential in the treatment of MM in preclinical studies by blocking triglyceride synthesis. However, early clinical trials have shown that DGAT1 inhibitors are prone to cause intolerable side effects such as gastrointestinal dysfunction and hypertriglyceridemia, which significantly limit their clinical application.^144^ Another type of DGAT2 inhibitor, PF-06424439, increases ceramide levels within the tumor cells, thus inducing leukemia cell death.^145^ Although both classes of DGAT inhibitors have shown anti-tumor prospects in preclinical models, the balance between efficacy and toxicity and the therapeutic window in specific disease contexts are still the core issues that need to be further explored in the future.

#### Targeting oxidative dependency: The role of CPT1 and FAO inhibitors in AML and stem cell maintenance

LSCs of AML are highly dependent on FAO to maintain their functional properties. This metabolic preference can maintain LSCs in a relatively quiescent state and enhance their ability to resist oxidative stress, thereby mediating chemotherapy resistance and promoting self-renewal. Therefore, inhibition of FAO is a potential strategy to eradicate LSCs and prevent disease recurrence.

##### *CPT*

CPT1, as the key rate-limiting enzyme in the FAO pathway, is the core target of this targeting strategy. As a CPT inhibitor, etomoxir can promote AML cell apoptosis by inhibiting the FAO of LSCs.^146^ However, its long-term application is prone to cause side effects such as oxidative stress, which limits its clinical transformation.^147^ In contrast, CPT1A-specific inhibitor ST1326 has the advantages of higher safety and reversible action, shows significant anti-tumor activity in the AML model, and can synergistically enhance the anti-leukemia effect of ABT199.^148^ Perhexiline, another CPT inhibitor, has shown strong anti-tumor potential and can effectively induce the apoptosis of CLL cells in the TME,^149^ highlighting its value in the treatment of malignant tumors under the protection of the TME, suggesting that FAO inhibitors have a good application prospect in hematological malignancies.

### Broad-spectrum strategies to regulate metabolic signaling hubs

Many hematological malignancies sustain FA metabolic rewiring through upstream regulatory hubs rather than a single enzymatic node, making these hubs attractive broad-spectrum vulnerabilities. The SREBP lipogenic program, PPAR-mediated FAO control, and PI3K-AKT-mTOR-driven growth–metabolism coupling collectively integrate genetic drivers and TME signals to maintain lipid supply, redox balance, and survival. Consequently, targeting these hubs can produce multi-pathway suppression and synergize with standard or immune-based therapies, while the degree of benefit is expected to be context-dependent and therefore requires subtype-informed application.

#### SREBP

SREBP is a central transcription factor that regulates the expression of genes involved in FA and cholesterol synthesis. In many hematological malignancies, the SREBP pathway is continuously activated to drive the lipogenic program and support tumor growth. Thus, SREBP inhibition is a broad-spectrum metabolic intervention. The small-molecule inhibitor fatostatin inhibits SREBP maturation and transcriptional activity by binding to SREBP cleavage-activating protein (SCAP) and preventing its transport from the ER to the Golgi.^150^ It shows therapeutic promise in primary cutaneous T-cell lymphoma, which is highly dependent on FAS.^151^ In addition, fatostatin can also exert an anti-proliferative effect by disrupting mitotic spindle assembly and inhibiting proliferation, inducing apoptosis, and regulating SREBP in drug-resistant ALL cells.^152^

#### PPAR

The PPAR family is the core transcription factor in lipid sensing and metabolism regulation, and its agonists have shown therapeutic potential in hematological malignancies by regulating FAO, lipid metabolism, and related signaling pathways. PPPARα and PPARγ agonists have shown anti-tumor activity by reducing lipid levels and promoting FAO. Fenofibrate and bezafibrate are important drugs in the clinical adjuvant treatment of hematological malignancies. Fenofibrate induces apoptosis in lymphoma and MM and reduces the risk of dexamethasone-induced osteonecrosis.^153^^,^^154^ In addition, bezafibrate combined with medroxyprogesterone acetate (BaP) has shown anti-cancer effects in AML.^155^ Recent studies have identified other PPARγ agonists that inhibit tumor cell proliferation, invasion, and angiogenesis.^14^ These compounds reduce the adhesion of HL-60 cells to vascular endothelial cells, modulate APL differentiation syndrome, and promote leukemic cell cycle arrest.^156^

#### mTOR

The mTOR pathway is a central hub that integrates growth factor signaling with cellular metabolism. Its abnormal activation is common in a variety of hematological malignancies, which promote protein and lipid synthesis and energy metabolism. Rapamycin and its derivatives are classical mTORC1 inhibitors. In AML and ALL, rapamycin can enhance the sensitivity of leukemia cells to chemotherapy by regulating mTORC1-mediated apoptosis and autophagy.^157^^,^^158^ In terms of immune regulation, mTOR inhibitors can attenuate the suppressive function of T cells and reduce antigen-and cytokine-induced B cell activation, thereby inhibiting excessive antibody production, which has the potential to treat related autoimmune phenomena.^159^^,^^160^ The PI3K–AKT–mTOR pathway is a key target for anti-cancer drug development, and novel PI3K/mTOR dual inhibitors, such as BEZ235, have shown significant anti-tumor activity and reversal of drug resistance in leukemia cells in preclinical studies.^161^

### Targeting FA metabolism combined with chemotherapy

Although drugs targeting FA metabolism cannot eliminate tumor cells, they can enhance the sensitivity of cells to radiotherapy and chemotherapy by blocking specific lipid pathways, effectively inhibiting the proliferation and metastasis of tumor cells.

FA uptake in lipid-rich TME dominated the proliferation of tumor cells. Depletion of CD36 could enhance the responsiveness of MCL cells to bortezomib and improve the therapeutic effect.^63^ At the same time, the change in lipid composition of the cell membrane will affect the efficiency of drug uptake. Inhibition of FASN can increase the proportion of polyunsaturated FA in the cell membrane and enhance membrane fluidity, thereby improving the sensitivity of tumor cells to chemotherapy drugs. FA metabolism inhibitor combined with venetoclax can produce a significant synergistic effect in the treatment of drug-resistant AML, and promote the apoptosis of chemotherapy-resistant cells.^162^^,^^163^ Energy metabolism adaptation is another important mechanism of tumor drug resistance. The expression level of CPT1 is increased in DLBCL patients with chemotherapy resistance, and the inhibition of CPT1 can improve the sensitivity of tumor cells to chemotherapy.^139^ Furthermore, the combination of FAO inhibitor and idelalisib produced a synergistic lethal effect on drug-resistant leukemia cells.^164^ A new cell death program can be induced by interfering with lipid storage and metabolic homeostasis. Inhibition of LD synthase DGAT1 enhances metformin-induced ferroptosis in AML.^165^ The mTOR inhibitor everolimus combined with imatinib can enhance the killing effect on CML cells by synergistically interfering with oncogenic signaling and metabolic reprogramming.^166^

These strategies collectively indicate that combining drugs targeting FA metabolism with chemotherapy can systematically disrupt the energy supply, membrane integrity, and survival signaling of drug-resistant cells, which is an effective direction for overcoming multidrug resistance.

### Targeting FA metabolism combined with immunotherapy

The reprogramming of FA metabolism in the TME not only supports tumor growth but also shapes the immunosuppressive microenvironment. Immunotherapy, represented by immune checkpoint blockade and adoptive cell therapy, aims to activate or enhance the ability of the immune system to recognize and kill tumor cells. The combination of targeted FA metabolism and immunotherapy can simultaneously weaken the metabolic advantages of tumor cells and improve the functional status of immune cells, thereby producing a synergistic anti-tumor effect.

The efficacy of inhibitors targeting programmed death 1 (PD-1), programmed death-ligand 1 (PD-L1), lymphocyte activation gene 3 (LAG-3), cytotoxic T-lymphocyte-associated protein 4 (CTLA-4), and other molecules is often limited by the lipid-rich immunosuppressive microenvironment in immune checkpoint blockade therapy. FA transporter CD36 is highly expressed in tumor cells and some immune cells, which can not only supply energy for tumor growth, but also regulate the functional adaptation of regulatory T cells in the TME through the PPARβ pathway to maintain immunosuppressive status.^167^^,^^168^ Blocking CD36 can reduce this immunosuppressive effect. Studies have found that CD36 and PD-1 are often highly expressed in extranodal lymphoma and other tumors. Combined use of CD36 inhibitors and anti-PD-1/PD-L1 antibodies has shown the potential to synergistically enhance anti-tumor immune responses in preclinical models,^169^ which provides a new direction for overcoming immune checkpoint inhibitor resistance.

Chimeric antigen receptor T-cell therapy (CAR-T) is an effective adoptive cell therapy for refractory hematological malignancies,^170^ but the core challenge is to maintain the persistence and functional activity of CAR-T cells in the metabolically unfavorable TME.^171^ The accumulation of FAs in the TME can reduce the activity of CAR-T cells. Therefore, strategies to modulate FA metabolism are being explored to enhance the function of therapeutic cells. In B-cell ALL models, inhibition of FA transporters to reduce intracellular lipid overload in CAR-T cells has been shown to improve their metabolic fitness, durability, and anti-tumor efficacy.^172^ These evidences suggest that combining CAR-T therapy with drugs targeting FA metabolism could improve therapeutic efficacy by improving the adaptation of CAR-T cells to TME and overcoming metabolic inhibition.

## Conclusions and future perspectives

Accumulating evidence demonstrates that FA metabolism reprogramming is not a uniform metabolic hallmark across hematological malignancies, but rather a disease- and context-specific adaptive program that underlies malignant survival, progression, and therapeutic resistance. Through coordinated rewiring of FAS, uptake, oxidation, and storage, malignant cells establish distinct metabolic dependencies that are tightly aligned with their proliferative state, cellular hierarchy, and microenvironmental constraints.

This review highlights that FA metabolic reprogramming in hematological malignancies follows a deterministic and hierarchical logic. Oncogenic drivers define the dominant metabolic direction, while transcriptional programs and signaling pathways progressively restrict metabolic plasticity. Persistent microenvironmental pressures then stabilize these adaptations into rigid metabolic dependencies. As a result, different disease entities converge on distinct FA metabolic nodes, such as synthesis-dominant programs in ALL and aggressive lymphomas, oxidation-dependent states in LSCs, or uptake- and storage-centered adaptations in chronic lymphoproliferative disorders and plasma cell malignancies. These dependencies are not merely correlative features, but functionally shape energy homeostasis, membrane organization, redox balance, cell fate decisions, and TME interactions.

Importantly, the functional consequences of FA metabolic reprogramming extend beyond supporting bioenergetic and biosynthetic demands. Altered FA metabolism actively reorganizes signaling networks, modulates susceptibility to oxidative stress and regulated cell death, promotes stress tolerance, and drives immune evasion and therapeutic resistance. By coupling metabolic state to signaling output and cell fate control, FA metabolism becomes a central determinant of malignant fitness rather than a passive metabolic adaptation.

From a translational perspective, these disease-specific metabolic dependencies represent predictable and targetable vulnerabilities. Targeting key nodes of FA metabolism can selectively disrupt malignant survival programs, sensitize tumor cells to chemotherapy, targeted therapy, and immunotherapy, and overcome resistance driven by metabolic plasticity. However, the pronounced heterogeneity of FA metabolic rewiring across hematological malignancies underscores the need for subtype-specific strategies rather than uniform metabolic interventions.

Future studies should further delineate how FA metabolism integrates with other metabolic networks, how microenvironmental cues dynamically shape metabolic dependencies, and how metabolic adaptations evolve under therapeutic pressure. Integrating multi-omics approaches with functional metabolic profiling will be essential to identify robust biomarkers of FA dependency and to guide precision metabolic interventions. Ultimately, leveraging the principles of metabolic vulnerability uncovered in FA metabolism holds promise for refining risk stratification, improving therapeutic efficacy, and advancing precision medicine in hematological malignancies.

## CRediT authorship contribution statement

**Yue Li:** Writing – original draft. **Tiange Lu:** Writing – review & editing. **Xiangxiang Zhou:** Writing – review & editing, Conceptualization.

## Funding

This work was supported by the 10.13039/501100001809National Natural Science Foundation of China (No. 82570247, 82170189), the Noncommunicable Chronic Diseases-10.13039/501100018537National Science and Technology Major Project (China) (No. 2025ZD0544300), the 10.13039/501100007129Shandong Provincial Natural Science Foundation (China) (No. ZR2025QC1702), and the Taishan Scholars Program of Shandong Province, China (No. tstp20250756).

## Conflict of interests

The authors declared no potential conflict of interests.
